# Is insufficient knowledge of epilepsy the reason for low levels of healthcare in the Lao PDR?

**DOI:** 10.1186/1472-6963-13-41

**Published:** 2013-02-04

**Authors:** Aina Harimanana, Phetvongsinh Chivorakul, Vimalay Souvong, Pierre-Marie Preux, Hubert Barennes

**Affiliations:** 1 Institut Francophone pour la Médecine Tropicale (IFMT), BP 9519, Vientiane, Lao PDR, Laos; 2 INSERM UMR 1094, Tropical Neuro epidemiology, Limoges, France; 3 School of Medicine, Institute of Tropical Neurology, Univ. Limoges, Limoges, France; 4 CHU Limoges, Limoges, France; 5 ISPED, Centre INSERM U897-Epidemiologie-Biostatistique, Univ. Bordeaux, F-33000, Bordeaux, France; 6 INSERM, ISPED, Centre INSERM U897-Epidemiologie-Biostatistique, F-33000, Bordeaux, France; 7 ANRS BP 983, Phnom Penh, Cambodia

**Keywords:** Epilepsy, Treatment gap, Knowledge, Medical staff, Nurse, Interventions

## Abstract

**Background:**

The treatment gap for epilepsy is considerable in low and middle-income countries. In the Lao PDR it is estimated at over 90%. Health workers play a significant role in bridging the gap between people with epilepsy (PWE) and access to epilepsy care. In a national survey we assessed: 1) the knowledge and practices of health workers in the Lao PDR towards the disease, and, 2) patient attendance at health facilities.

**Methods:**

We conducted a random three-stage sampling of health workers at the provincial, district and health center levels in 2009.

**Results:**

Overall, 284 health workers were enrolled in 50 health facilities of 11 provinces: health centers 24.7%; district hospitals 23.2%; and province hospitals 52.1%. Only a minority of these (2.5%) recalled ever having received training or seeking information on epilepsy. Our survey showed a lack of knowledge in diagnosing and prescribing drugs for epilepsy, including phenobarbital, the first-line of treatment. The majority of respondents (59.9%) was unaware of the availability of antiepileptic drugs in health facilities. Only 10 (20%) health facilities, and no health centres, received people with epilepsy. It was estimated that one PWE per month receives medical attention. Traditional beliefs about PWE were common; such as the idea that epilepsy could be transmitted through saliva (63.2%). A higher attendance of PWE was observed in province hospitals where the knowledge of epilepsy care was higher. Global acceptance of people with epilepsy was low.

**Conclusions:**

The low level of knowledge of epilepsy on the part of health workers may be contributing to the wide treatment gap in the Lao PDR. Improving knowledge of this disease and increasing the availability of antiepileptic drugs will reduce misconceptions about epilepsy, thus encouraging more PWE to seek treatment. Community-based educational programs and extensive advocacy for people with epilepsy only began in 2009.

## Background

In developing countries, general physicians and nurses play a major role in providing medical care and social support to people with epilepsy (PWE) [1,2]. Health staff educates PWE and their relatives to enhance motivation and confidence in taking antiepileptic drugs and to decrease the level of stigma [3]. Nurses and non-medical health workers are often the only health staff available to diagnose PWE [4]. To be effective, they usually receive specific short or long term training on epilepsy [5,6]. In countries where no specific programs on epilepsy exist, health centres with available health workers and essential drugs must suffice to take care of PWE. However, little data is available on the specific knowledge of the health staff from different settings or specialties [6]. In the Lao People’s Democratic Republic (Lao PDR), medical health staff qualified and knowledgeable on the treatment of epilepsy is scarce and mostly based in the capital: 1 neurologist; 2 psychiatrists; and 1 neurosurgeon [7]. Patients mainly attend the psychiatric department of Mahosot Hospital in Vientiane Capital [8]. In addition to the services available in Vientiane, 4 psychiatric wards have mental health and epilepsy inpatient care and are theoretically available in 4 provinces (Khamuan, Luang Prabang, Udomxai, Savannaket) following short training in 2000. Misconceptions about epilepsy are prevalent in the community with frequent shortages of anti-epileptic drugs (AED). The treatment gap is estimated to be more than 90% [7,9].

The poor knowledge of health staff has been suggested as a contributing cause to the epilepsy treatment gap [10]. Proper knowledge of health staff is difficult to evaluate and was rarely assessed. The objective of this study was to report on the medical staff knowledge of epilepsy in the Lao PDR, and the potential implication on the epilepsy treatment gap.

## Methods

### Study setting

The Lao PDR was ranked 122 out of 179 nations on the Human Development Index in 2010 [11]. Although it is a land-locked country, the opening of roads and borders enables Lao citizens to seek healthcare abroad [12]. Over 52 000 PWE were estimated to live in the country [13].

Overall, the Lao PDR has 905 health facilities, distributed as follows: 4 reference hospitals (RH); 3 specialized hospitals (dermatology, ophthalmology, rehabilitation) in Vientiane Capital; 16 provincial hospitals (PH); 125 district hospitals (DH); and 757 health centers (HC). Patients are theoretically expected to consult at the lowest level community health centers, which are run by either a general practitioner or a nurse. If the patient needs specialized medical treatment, he/she is referred to a higher level and so forth. Health facilities are underutilised. In 2001, a Ministry of Health report established that 1.9% of patients seek care at the health centers, 4.9% at PH, and 7.1% at DH; 18.7% went directly to the pharmacy without seeing a health worker (HW), 52.9% reported a history of self-medication, and 1.0% went to traditional healers [14].

Based on the level of training and existing curricula, three types of HW exist in the Lao PDR: i) low level HW (low level auxiliary nurses) are trained at the College of Health Technology for two and a half years; ii) middle level HW (medical assistants, pharmacists assistants, high level nurses) follow a three-year curriculum in nursing schools; and iii) high level HW (medical doctors, MSc, pharmacists) graduates from the Faculty of Medical Science and Pharmacy after five years of training. The ratio of medical staff (1.59 per 1000 inhabitants) is below the international standards [12]. The distribution of HWs is uneven across the country: Health centers are almost totally served by low (81%) and mid-level (18%) staff. Physicians are concentrated in urban areas [12].

Training of physicians in epilepsy is limited to 4 weeks training in the psychiatric ward of Mahosot Hospital (Vientiane) as part of the curricula during medical university.

Nurses attend no lectures.

### Study design and procedures

A random three-stage sampling study was conducted from February to March 2009. During the first stage, 11 province hospitals (PH) out of 18 provinces were selected using a random number table. The second stage involved a random selection of one district hospital (DH) per province. The third stage involved two health centres (HC) per district or a third HC if the district had no DH. Health staff were included if they provided clinical care for adult patients, had held their current position for at least one year, and were able to volunteer one hour to the survey. At the health centre level, all the available medical health staff was included. The referral hospitals were not included in the study. At the end of each interview a book on “Caring for PWE” was provided to the respondents.

### Survey questionnaire

A 55-item questionnaire was designed, based on other survey instruments, to assess the knowledge of health staff, their attitudes, beliefs and practices toward epilepsy [1,15]. The questionnaire, in Lao language, elicited information on: demographics (5); personal experience with epilepsy (4); epilepsy care knowledge; diagnosis (12); treatment (27); and social considerations toward PWE (7). The number of PWE consulting health facilities was drawn from the health facility records of the last 2 months and its accuracy was checked with the health staff.

The questionnaire was pre-tested on a pilot group of health staff for accuracy and comprehension. The questionnaire was self-administered. The study team supervised the survey, obtained the trust of all respondents by assuring their anonymity, and made the respondents feel comfortable in expressing their views. A feedback form, including main pitfalls found during the survey, was sent to the respondents after the survey.

The ILAE definition of epilepsy: recurrence of at least two spontaneous seizures during an interval of at least 24 hours (ILAE, 1993); and the 1981 ILAE classification into partial seizures or generalised seizures was used (ILAE, 1981) as a reference answer.

Medical health workers were categorized in two groups: nurses--for all types of nurses; and physicians--for medical doctors and medical assistants.

### Data management and analysis

Data was processed using Epidata ( http://www.epidata.dk, Odense, Denmark) and Stata, Version 8 (Stata Corp., College Station, TX, USA). Chi-square and Fisher’s exact tests for categorical variables, and Student’s test and analysis of variance (F test) for normally distributed continuous data, were used for data analysis. The threshold for statistical significance was preset at 0.05. Analysis was conducted within the two categories (nurses or physicians) than according to the type of health facility. The results were pooled if no significant difference was observed within a category. Results are presented according to the qualifications and the type of health facilities.

### Sample size

Using Stata Version 8 (Stata Cooperation, College Station, TX), a needed sample size of 295 people was established, based on a previous estimate of 26% for misconceptions of epilepsy as a communicable disease among physicians working in paediatric wards, 5% precision, alpha = 0.05, and 10% of anticipation for drop-outs or refusals [8].

### Ethical approval

Ethical clearance was obtained from the Lao National Ethics Committee and informed written consent from the health staff. Answers of the health staff were kept confidential.

## Results

### Characteristics of the health staff

The study was conducted in 50 health facilities in March 2009: 11 province hospitals; 9 district hospitals; and 30 health facilities. In total 304 health staff were enrolled. None of the health staff enrolled refused to take part in the study. Twenty, who had no clinical activity, or had only administrative function, were excluded leaving 284 medical health staff (140 nurses, 144 physicians) for analysis. Overall 70 (24.7%) worked in HC, 66 (23.2%) DH, and 148 (52.1%) in PH. The main characteristics of health staff and their experience with epilepsy are shown Table 1. A minority of respondents recalled any training received (2.5%), or ever searched for information (22.9%) on epilepsy.

**Table 1:** Medical health workers’ characteristics and previous experience with epilepsy in Lao PDR (2009)

	**Nurses**	**Physicians**	***p***	**Total**
	**n = 140 (50.7%)**	**n = 144 (49.3%)**		**n = 284 (%)**
Sex (Male)	18 (12.4)	48 (33.3)	<0.001	66 (23.2)
Over 10 years in current position	57 (40.7)	68 (47.2)	0.2	125 (44.0)
Number of years in the ward *	1.1 (1.1-1.2)	1.1 (1.1-1.2)	0.5	1.1 (1.1-1.2)
Number of years since graduation*	14.2 (12.7-15.8)	14.4 (12.8-15.9)	0.9	14.3 (13.2-15.4)
Knows a PWE	31 (22.1)	26 (18.1)	0.4	57 (20.1)
Has diagnosed epilepsy	16 (11.4)	58 (40.3)	<0.001	74 (26.1)
Has received extra-training in epilepsy	2 (1.4)	5 (3.5)	0.4	7 (2.5)
Has searched information about epilepsy	20 (14.3)	45 (31.3)	0.001	65 (22.9)
Thinks that no AED is available in his/her health facility	69 (49.3)	101 (70.1)	<0.001	170 (59.9)
Trusts in AED treatment	59 (42.1)	68 (47.2)	0.4	127 (44.7)

* mean (CI 95%).

*PWE* = People With Epilepsy, *AED* = antiepileptic drug.

### Knowledge and practices towards diagnosis and prognosis of epilepsy

**Figure 1:**
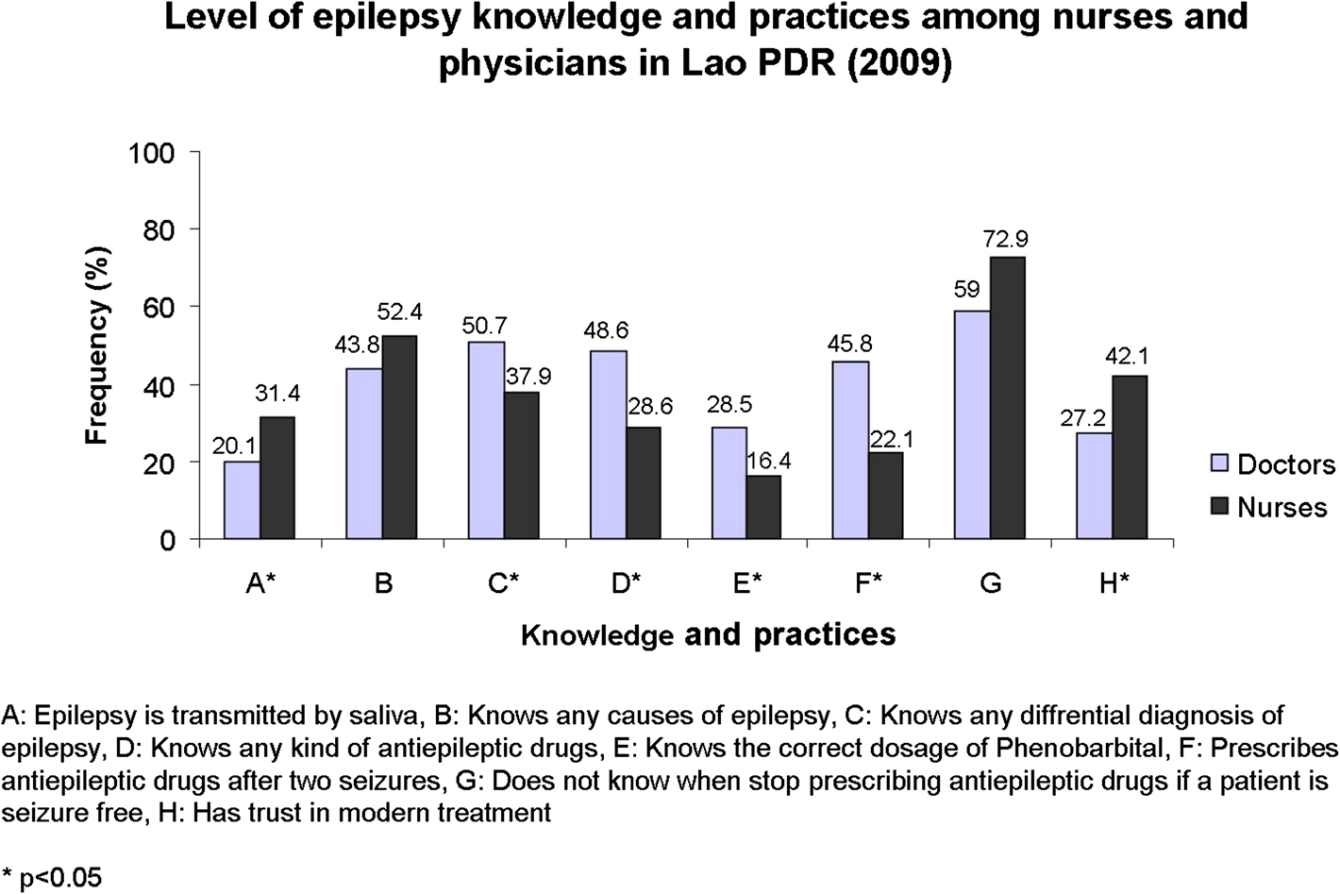
Level of epilepsy knowledge and practices among nurses and physicians in Lao PDR (2009).

Overall, nurses had lower knowledge and wrong practices toward epilepsy compared to physicians (Figure 1). Only 74 HWs (26.1%) had ever diagnosed epilepsy. None of the respondents could give a proper definition of epilepsy. Of 284 HW, 23 (15.9%) could classify epilepsy and 117 (41.2%) considered it a communicable disease transmitted mostly by saliva (63.2%) or genes 45 (15.9%). The majority (52.8%) could not describe the most frequent type and causes of seizures. None cited supernatural causes for epilepsy. The prognosis of PWE was unknown for 198 (69.7%), considered as severe for 47 (16.6%), or lethal for 18 (6.3%). Only 21 (7.4%) considered that it could be cured.

### Knowledge and practices towards antiepileptic drugs

The majority of the respondents (59.9%) were unaware of any available AED at the health facilities. Many did not know the names of any AED (57.3%) and 47.5% did not know precisely when to start prescribing AEDs. Of 284, 34.1% would prescribe one AED to a patient with a monthly seizure. Only 28 (9.9%) of HW would prescribe a long-term treatment. Of the 74 HWs (26.1%) who had ever diagnosed epilepsy, only 50 (71.1%) had prescribed an AED. Phenobarbital was known by 121 (42.6%) and intended to be prescribed alone (25.0%) or associated either with Diazepam (2.5%), or Valproic acid (1 physician). Mistrust (55.3%) of AEDs was reported at similar frequencies among nurses and physicians.

Physicians from province hospitals had better knowledge and practices toward diagnosis and use of AEDs. Details on epilepsy knowledge care are given in Table 2.

**Table 2:** Physicians’ knowledge attitude and practices about epilepsy according to health facilities in Lao PDR (2009)

	**Province Hospital**	**District Hospital/Health Centers**	**Total**
	**n = 63 (43.7%)**	**n = 81 (56.3%)**	**n = 144**
**Knowledge**			
Epilepsy is a communicable disease *	27 (42.9)	21 (25.9)	48 (33.3)
- Transmitted by saliva	15 (55.6)	14 (66.7)	29 (60.4)
- Transmitted by genes	10 (15.9)	11 (13.6)	21 (14.6)
Does not know any causes of epilepsy*	26 (41.3)	55 (67.9)	81 (56.3)
Diagnosis needs EEG or CT scan *	14 (22.2)	42 (51.9)	56 (38.8)
Cannot describe or classify seizures *	51 (81.0)	79 (97.5)	130 (90.3)
Knows one differential diagnosis of E *	33 (52.4)	28 (34.6)	61 (42.4)
Knows more than one long term AED *	34 (54.0)	14 (17.3)	48 (33.3)
Knows at least one AED *	51 (81.0)	28 (34.6)	79 (54.9)
- Knows Phenobarbital alone *	29 (46.0)	11 (13.6)	40 (27.8)
- Does not know the correct doses of PB *	25 (39.7)	16 (19.8)	41 (28.5)
- Knows Diazepam alone	6 (9.5)	4 (4.9)	10 (6.9)
- Does not know the correct doses of diazepam	4 (6.4)	5 (6.2)	9 (6.3)
Aware that discontinuing AED is life threatening*	46 (73.0)	33 (40.7)	79 (54.9)
**Practice**			
Prescription in case of overt epilepsy			
- Prescribe AED *	41 (65.1)	25 (30.9)	66 (45.8)
- Prescribe Phenobarbital alone *	34 (54.0)	14 (17.3)	48 (33.3)
- Prescribe Diazepam alone	4 (6.4)	6 (7.4)	10 (7.0)
- Prescribe a polytherapy	3 (4.8)	5 (6.2)	8 (5.6)
Number of seizures before prescribing AED			
- Does not know *	16 (25.4)	45 (55.6)	61 (42.4)
- After one or more seizures	16 (25.4)	18 (22.2)	34 (23.6)
- After two or more seizures *	23 (36.5)	10 (12.4)	33 (22.9)
- After 3 or more seizures	5 (7.9)	7 (8.6)	12 (8.3)
Duration of AED treatment			
- Does not know *	44 (69.8)	75 (92.6)	119 (82.6)
- Less than one month	3 (4.8)	5 (6.2)	8 (5.6)
- One month to one year *	9 (14.3)	0	9 (6.3)
- More than two years *	7 (11.1)	1 (1.2)	8 (5.6)
Time to discontinue AED in seizure-free patients			
- Does not know *	29 (46.0)	56 (69.1)	85 (59.0)
- Between 1 to 2 years	21 (33.3)	16 (16.8)	37 (25.7)
- After 2 years	6 (9.5)	4 (4.9)	10 (6.9)
- Never	7 (11.1)	5 (6.2)	12 (8.3)
Give any advice to PWE *	57 (90.5)	47 (58.0)	104 (72.2)
- Explain about the disease	1 (1.6)	1 (1.2)	2 (1.4)
- Observance *	25 (39.7)	15 (18.5)	40 (27.8)
- Avoid risk factors *	44 (69.8)	38 (49.9)	82 (56.9)
- Avoid danger *	43 (68.3)	37 (45.7)	80 (55.6)

* p < 0.05.

*AED*: antiepileptic drugs, *CT*: Computerized Tomography, *E*: epilepsy, *EEG*: electroencephalography, *PB*: Phenobarbital, *PWE*: People With Epilepsy.

### Social acceptance of PWE

Fear of epilepsy and the social acceptance of the disease were similar between nurses and physicians. The acceptance rate of each item referring to the perception ranged from 55% to 83% for all criteria except for the item related to marriage opportunities (27.8%). Physicians of major hospitals (PH) had the highest social acceptance of the disease (Table 3).

**Table 3:** Social attitude of health workers toward PWE in Lao PDR (2009)

**Answer (yes)**	**Nurses**	**Physicians**	***p***	**Total**
	**n = 140 (49.3%)**	**n = 144 (50.7%)**		**n = 284 (%)**
**Do you think that PWE:**				
Are accepted by Lao society?	91 (65.0)	92 (68.4)	0.8	183 (64.4)
Can study in the same way as their peers?	94 (67.1)	109 (75.7)	0.1	203 (71.5)
Cando the same work as their peers?	83 (59.3)	88 (61.1)	0.8	171 (60.2)
Can have a normal life with treatment?	102 (72.9)	120 (83.3)	0.03	222 (78.2)
**Will you let a PWE:**				
Marry your child?	37 (26.4)	42 (29.2)	0.6	79 (27.8)
Play with your child?	77 (55.0)	96 (66.7)	0.04	173 (60.9)
**Afraid of PWE**	40 (28.6)	41 (28.5)	1.0	81 (28.5)

*PWE*: People With Epilepsy.

### PWE attendance

Of 50 health facilities, none had a specific epilepsy service. Half of the PH and DH officially reported a consultation for clinical care of PWE, where AED are supposed to be available. In fact, 10 (25%) health facilities, 7 province hospitals and 3 district hospitals) had received one or more PWE only, totaling 40 PWE in the prior 2 months. Of the 40 PWE, 24 (60%) came on a routine basis, and 16 (40%) were seen during an emergency. No PWE came for consultation to any of the 30 HC. In total 26 PWE [mean: 2.3 (95% CI: 1.3-3.2)] and 14 PWE [mean: 1.7 (95% CI: 0.5-2.8)] were reported in the previous two months in the PH and DH, respectively. A crude extrapolation suggests that a mean of one PWE was being cared per hospital per month in Lao PDR with 40% in the context of an emergency.

## Discussion

This nationwide survey documented a lack of knowledge of health care personnel about epilepsy and the poor access of PWE to AEDs and health facilities in the Lao PDR.

Concerning the knowledge of epilepsy care, 3 main results can be pointed out. 1) Medical health staff’s global knowledge of epilepsy was low. Compared to studies conducted among health staff students in Cameroon, our findings showed a higher number of respondents who did not know any cause of epilepsy, or who thought that epilepsy was a communicable disease [16,17]. 2) Personal concerns toward PWE were high among medical health staff. The majority of our respondents were afraid of a PWE. Fewer health staff would allow their children to marry a PWE compared to results from Cameroon, or from the community level in the Lao PDR [1,7,16]. However, a similar percentage of respondents would allow PWE to achieve the same work of their peers in Cameroon and Lao PDR [17]. 3) The knowledge of prescribing AED is scarce among the health staff. A low trust in AED treatment, and misconceptions that epilepsy is incurable was described. In the Lao PDR, little time is devoted to epilepsy in the medical or nursing curricula. The shortage of medical books has been a major problem during the last 25 years in the Lao PDR. The relatively low proportion of respondents who had ever read about epilepsy confirmed this. Consistent with a previous study conducted among health staff in pediatric wards, these findings suggests that traditional beliefs of the Lao population are commonly shared by the medical staff [8]. A call for action is then needed for epilepsy: (A) Enhancing initial training and postgraduate refinement of education regarding epilepsy. (B) Addressing the negative attitudes and discrimination through a global campaign on epilepsy in the Lao PDR. Families of PWE who have been shown to have fewer stigmas than general population might help to improve social acceptance of PWE [7]. (C) Implementing Lao national guidelines for the management of PWE. A similar situation was successfully addressed through dedicated programming in Thailand [18].

PWE attendance at health facilities is low. Our findings suggest that the quality of care offered to PWE is limited to simple instructions or to the *ad hoc* treatment of a seizure, which does not help in proper treatment of the illness. The distance between health care facilities and PWE and scarce availability of AED also contribute to low attendance [19,20]. The poor level of health staff knowledge and awareness of epilepsy may be determining the low utilization of health care facilities by PWE.

Bridging the gap between PWE and health facilities is a major issue that can only be overcome with a dramatic improvement of health staff knowledge [21,22]. Since the launching of Initiative for Epilepsy in the Lao PDR, activities aiming at improving health staff knowledge are currently being carried out [23,24]. Since 2010, specific 3-day epilepsy training with ongoing clinical epilepsy support has been provided to over 80 medical health staff in 30 districts of 6 provinces by the Initiative (around 20% of all districts in Lao PDR). Two large surveys to screen PWE involving over 40 medical students were conducted in 2009 and 2011 [23]. Postgraduate training in the Lao PDR has been informal until recently [8]. Epilepsy is now on the regular agenda of the annual paediatrics meeting and lectures are now more frequent at medical schools and institutions. The status of a Lao Medical Association against Epilepsy, one of the first medical associations being founded in the country was transmitted for agreement to the government in 2011. Efforts are now being conducted to emphasize the understanding of the disease and to promote knowledge of its curability to the population. This includes a daily radio spot, leaflets in Lao language for patients, and guidelines for physicians [23]. An evaluation of these interventions should be planned.

Evaluating the knowledge levels of health staff, of a highly neglected and stigmatised disease, was considered a difficult challenge. Staff, who is supposed to care for PWE, may be reluctant to report knowledge deficits. This was resolved by the choice of a self-questionnaire which allowed them to answer directly but without the risk of embarrassment or negative consequences. Furthermore, the team study tried to make interviewees comfortable by providing clear explanations of the survey before giving the questionnaire. However, some open questions; such as fear of patients, reasons of low access, and explanations of poor knowledge of epilepsy require further study.

Having excluded the referral hospitals in Vientiane Capital may constitute a selection bias that has probably a minor effect considering that only four physicians are providing medical care to PWE in these hospitals. Excluding the referral hospital would probably not affect the accuracy of our results.

## Conclusion

Knowledge about epilepsy is poor among health staff in the Lao PDR, particularly among nurses and physicians working at primary and secondary levels such as DH and HC. The first point of care for PWE is infrequently accessed. Basic and continuing interventions to increase knowledge of epilepsy and its treatment must be provided to health staff to reduce the stigma towards PWE. This should be advocated strongly and must be combined with information to the general population about the treatability of the disease. This will ensure improvement in the condition of PWE in the Lao PDR.

## Abbreviations

AED: Anti-Epileptic Drugs; DH: District Hospital; HC: Health Center; HW: Health Worker; IFMT: Institut Francophone pour la Médecine Tropicale; FITM: (Francophone Institute for Tropical Medicine); ILAE: International League Against Epilepsy; Lao PDR: Lao People’s Democratic Republic; PWE: People With Epilepsy; PH: Province Hospital.

## Competing interests

PC and VS received a monthly allocation from Initiative on Epilepsy in Laos whose funds were originally supported by SANOFI AVENTIS Access to Drugs. AH was granted fellowship from Conseil Régional de Limoges France. None of the funders had any responsibility in the survey, its analysis and in the decision to publish.

## Authors’ contributions

HB is the principal investigator of the study. HB, AH designed the survey and coordinated data acquisition. HB, AH, PMP made substantial contributions to conception and design of the manuscript, were involved in statistical analyses and interpretation of the data. VS and PC contributed to data collection and comments on the manuscript. All authors read and approved the final manuscript.

## Pre-publication history

The pre-publication history for this paper can be accessed here:

http://www.biomedcentral.com/1472-6963/13/41/prepub

## References

[B1] Chomba EN Haworth A Atadzhanov M Mbewe E Birbeck GL Zambian health care workers’ knowledge, attitudes, beliefs, and practices regarding epilepsy Epilepsy Behav 2007 10 111 119 10.1016/j.yebeh.2006.08.012 17055341 PMC2938019

[B2] Elliott J Shneker B Patient, caregiver, and health care practitioner knowledge of, beliefs about, and attitudes toward epilepsy Epilepsy Behav 2008 12 547 556 10.1016/j.yebeh.2007.11.008 18171634

[B3] Hawley SR Paschal AM Ablah E St RT Liow K Molgaard CA Initial perspectives from Midwestern neurologists: epilepsy patients’ barriers and motivators for seeking treatment Epilepsia 2007 48 1920 1925 10.1111/j.1528-1167.2006.01137.x 17561955

[B4] Adamolekun B Mielke J Ball D Mundanda T An evaluation of the management of epilepsy by primary health care nurses in Chitungwiza. Zimbabwe Epilepsy Res 2000 39 177 181 10.1016/S0920-1211(99)00115-1 10771243

[B5] Fernandes PT Noronha AL Sander JW Bell GS Li LM Training the trainers and disseminating information: a strategy to educate health professionals on epilepsy Arq Neuropsiquiatr 2007 65 Suppl 1 14 22 17581663 10.1590/s0004-282x2007001000003

[B6] Locharernkul C Suwaroporn S Krongthong W Limarun C Arnamwong A A study of knowledge and attitude improvement on epilepsy among Thai physicians and nurses J Med Assoc Thai 2010 93 875 884 20718161

[B7] Tran DS Odermatt P Singphuoangphet S Druet-Cabanac M Preux PM Strobel M Epilepsy in Laos: knowledge, attitudes, and practices in the community Epilepsy Behav 2007 10 565 570 10.1016/j.yebeh.2007.02.018 17446140

[B8] Barennes H Sengkhamyong K Sambany EM Koffi PN Chivorakul P Empis G Children’s access to treatment for epilepsy: experience from the Lao People’s Democratic Republic Arch Dis Child 2011 96 309 313 10.1136/adc.2009.181255 20810400

[B9] Barennes H Tran DS Latthaphasavang V Preux PM Odermatt P Epilepsy in Lao PDR: From research to treatmentintervention Neurology Asia 2008 13 27 31

[B10] Meinardi H Scott RA Reis R Sander JW The treatment gap in epilepsy: the current situation and ways forward Epilepsia 2001 42 136 149 11207798 10.1046/j.1528-1157.2001.32800.x

[B11] United Nations Development Program Human development report 2010 2010 http://hdr.undp.org/en/reports/global/hdr2010/

[B12] WHO Human Resources for Health: Analysis of the Situation in Lao PDR; WPRO 2007 Country Assessment Profiles, Lao People’s Democratic Republic

[B13] Tran DS Odermatt P Le TO Huc P Druet-Cabanac M Barennes H Prevalence of epilepsy in a rural district of central Lao PDR Neuroepidemiology 2006 26 199 206 10.1159/000092407 16569936

[B14] Ministry Of Health National institute of public health Report on national health survey 2001 Health status of the People in Lao PDR http://books.google.fr/books/about/Health_status_of_the_people_in_Lao_P_D_R.html?id=A_LaAAAAMAAJ&redir_esc=y

[B15] Doshi D Reddy BS Kulkarni S Karunakar PNA Dentists’ knowledge, attitudes and practices toward patients with epilepsy in Hyderabad city, India Epilepsy Behav 2012 23 447 450 10.1016/j.yebeh.2012.01.022 22381393

[B16] Njamnshi AK Tabah EN Bissek AC Yepnjio FN Angwafor SA Dema F Knowledge, attitudes and practices with respect to epilepsy among student nurses and laboratory assistants in the South West Region of Cameroon Epilepsy Behav 2010 17 381 388 10.1016/j.yebeh.2009.12.027 20153701

[B17] Njamnshi AK Angwafor SA Baumann F Angwafo FF III Jallon P Muna WF Knowledge, attitudes, and practice of Cameroonian medical students and graduating physicians with respect to epilepsy Epilepsia 2009 50 1296 1299 10.1111/j.1528-1167.2009.02155.x 19496813

[B18] Tiamkao S Tiamkao S Auevitchayapat N Arunpongpaisal S Chaiyakum A Jitpimolmard S Basic knowledge of epilepsy among medical students J Med Assoc Thai 2007 90 2271 2276 18181306

[B19] Chivorakul P Harimanana AN Clavel S Jousseaume S Barennes H Epilepsy in Lao PDR: the uneasy procurement of the first line antiepileptic contributes to the high treatment gap understanding Rev Neurol 2011 In press 10.1016/j.neurol.2012.01.584 22405460

[B20] Odermatt P Ly S Simmala C Angerth T Phongsamouth V Mac TL Availability and costs of antiepileptic drugs and quality of phenobarbital in Vientiane municipality. Lao PDR Neuroepidemiology 2007 28 169 174 10.1159/000103270 17536229

[B21] Li SC A report on a feasibility test of “community control of epilepsy” proposed by WHO Zhonghua Shen Jing Jing Shen Ke Za Zhi 1989 22 144 147 190 2512104

[B22] Guinhouya KM Aboki A Kombate D Kumako V Apetse K Belo M The epilepsy treatment gap in six primary care centres in Togo (2007–2009) Sante 2010 20 93 97 20682482 10.1684/san.2010.0193

[B23] Barennes H Chivorakul P Groupe de travail Epilepsie 2011 http://www.ifmt.auf.org/rubrique.php3?id_rubrique=62

[B24] Chivorakoun P Harimanana A Clavel S Jousseaume S Barennes H [Epilepsy in Lao popular democratic republic: difficult procurement of a first-line antiepileptic contributes to widening the treatment gap] Rev Neurol (Paris) 2012 168 221 229 10.1016/j.neurol.2012.01.584 22405460

